# National evaluation of strategies to reduce safety violations for working from heights in construction companies: results from a randomized controlled trial

**DOI:** 10.1186/s12889-016-2693-x

**Published:** 2016-01-09

**Authors:** Henk F. van der Molen, Aalt den Herder, Jan Warning, Monique H.W. Frings-Dresen

**Affiliations:** 1 Coronel Institute of Occupational Health, Academic Medical Center, University of Amsterdam, P.O. Box 22660, 1100 DD Amsterdam, The Netherlands; 2 Arbouw, P.O. Box 213, 3840 AE Harderwijk, The Netherlands

**Keywords:** Evaluation, Safety hazards, Working from heights, Safety behavior, Construction industry

## Abstract

**Background:**

The objective of this study is to evaluate the effectiveness of a face-to-face strategy and a direct mail strategy on safety violations while working from heights among construction companies compared to a control condition.

**Methods:**

Construction companies with workers at risk for fall injuries were eligible for this three-armed randomized controlled trial. In total, 27 cities were randomly assigned to intervention groups–where eligible companies were given either a face-to-face guidance strategy or a direct mailing strategy with access to internet facilities–or to a control group. The primary outcomes were the number and type of safety violations recorded by labor inspectors after three months. A process evaluation for both strategies was performed to determine reach, program implementation, satisfaction, knowledge and perceived safety behavior. A cost analysis was performed to establish the financial costs for each intervention strategy. Analyses were done by intention to treat.

**Results:**

In total, 41 % (n = 88) of the companies eligible for the face-to-face intervention participated and 73 % (n = 69) for direct mail. Intervention materials were delivered to 69 % (face-to-face group) and 100 % (direct mail group); completion of intervention activities within companies was low. Satisfaction, increase in knowledge, and safety behavior did not differ between the intervention groups. Costs for personal advice were 28 % higher than for direct mail. Ultimately, nine intervention companies were captured in the 288 worksite measurements performed by the labor inspectorate. No statistical differences in mean number of safety violations (1.8–2.4) or penalties (72 %–100 %) were found between the intervention and control groups based on all worksite inspections.

**Conclusions:**

No conclusions about the effect of face-to-face and direct mail strategies on safety violations could be drawn due to the limited number of intervention companies captured in the primary outcome measurements. The costs for a face-to-face strategy are higher compared with a direct mail strategy. No difference in awareness and attitude for safe working was found between employers and workers between both strategies.

**Trial registration:**

NTR 4298 on 29-nov-2013.

## Background

Construction workers are frequently exposed to various types of injury-inducing hazards, especially falling from heights [1–3]. Most fall accidents from scaffolds can be prevented through compliance with regulations [2]. Psychosocial factors can also contribute to occupational accidents. For the construction industry, high time pressure and exposure to violence and harassment by colleagues or supervisors are associated with occupational accidents [4]. Both organizational and project levels are reported as being important for promoting safety performance; leadership and commitment are important at the organization level, while risk assessment and management are important at the project level [5].

More than 80 % of Dutch construction sites violate safety regulations for working from heights (personal communication), while 14 % of the construction workers report that unsafe situations regularly prevail at worksites [6]. Specifically concerning small companies in the construction industry, there seems to be a lack of information on hazard recognition [7]. To increase compliance with safety procedures, employers and workers need to select, implement, and monitor safety measures. To facilitate this behavioral change [8], stimulating knowledge awareness [9] and personalized feedback [10, 11] are behavior change techniques that are frequently advocated. In addition, education and subject matter training could improve occupational safety and health of the small business workforce in the residential construction industry [7]. The involvement of unions, employers’ organizations (e.g. [12]) and the labor inspectorate (e.g. [13]) is recommended when executing national programs to improve safety and health at job, company or branch level.

For this study, two behavior change strategies were developed, based on aspects of awareness-raising and personalized feedback. These consisted of: 1) face-to-face contacts with safety consultants; and 2) direct mail with internet links. We hypothesize that both guidance strategies will reduce safety violations with rolling scaffolds, ladders and stairs compared to the control condition of a general announcement of inspection in construction companies. In addition, the face-to-face strategy is thought to be superior to the direct mail strategy due to a higher impact of personalized feedback. The financial costs are expected to be higher in the face-to-face guidance strategy, especially due to hiring safety consultants and their coordinator for worksite visits.

The objective of this study is to evaluate the effectiveness of a face-to-face strategy and a direct mail strategy on safety violations for working from heights among construction companies compared to the control condition of only announcing safety inspections by the labor inspectorate. For both guidance strategies, a process and cost evaluation will be performed.

## Methods

### Design

A three-armed randomized controlled trial (RCT) was performed to compare the effectiveness of two behavior change strategies: a face-to-face guidance strategy and a direct mailing strategy, with a control condition. For the description of the design of the safety intervention and the two guidance strategies, in compliance with the CONSORT statement, we refer to [14]. No changes to methods or outcomes after trial reporting [14] and commencement occurred. The reporting of this study adheres to the CONSORT guideline for reporting randomized trials.

In addition to the RCT, a process evaluation and a cost evaluation took place for each guidance strategies. In total, 27 large cities were stratified within three regions in the Netherlands (North-East, West, South) and each city was assigned to one of two intervention groups or the control group using nQuery Advisor® Version 7.0. As a result of the inspection procedure of the Dutch Labor Inspectorate, i.e. unannounced worksite inspections in a well-defined area and time period in the Netherlands, the interventions took place at construction companies working in larger cities. The study protocol did not meet the criteria of the “Medical-scientific research with human participants Act”, i.e. it was not a study of a medical nature and the subjects do not receive a particular treatment or are asked to behave in a particular way [15]. The ethics committee of the Academic Medical Center in Amsterdam confirmed that the study did not meet the criteria of the ‘Medical-scientific research with human participants Act’ and therefore no ethics approval was required. For the full description of the design of the safety intervention and the two guidance strategies, we refer to [14].

### Subjects

The guidance strategies were given to construction companies. Inclusion criteria of the construction companies were: 1) involved in the painting and maintenance of buildings; and 2) working in one of the 18 pre-randomized bigger cities in the Netherlands during May 2014.

Construction companies willing to participate were contacted in December 2013/January 2014 for further arrangements concerning the proposed interventions, and employers and workers were formally asked for their consent when sending completed questionnaires. The research population included construction sites of the participating companies. With the exception of a general national announcement of inspection in construction companies, no guidance strategies took place in the control group. Allocation occurred on the basis of eligibility, i.e. the companies that were assumed to have construction projects in one of the randomized cities during May 2014 (four to five months after allocation). The researcher (HM) assigned companies to the interventions, while a call center enrolled eligible companies.

### Interventions

The safety measures covered by the two guidance strategies were based on the inspection module of the labor inspectorate and the guidelines of the Dutch construction safety and health institute Arbouw. For this study, two behavior change strategies were developed in collaboration with employers’ organizations and unions to reduce the number of safety violations during the installation and use of rolling scaffolds, ladders and stairs among painters, in addition to the announcement of safety inspections by the labor inspectorate (interventions are described in detail in [14]). No blinding for the assignment of interventions was possible.

#### Face-to-face guidance strategy

The face-to-face guidance strategy consisted of personal advice at construction companies with a maximum of three visits during which workers were informed about preventing falling hazards with rolling scaffolds, ladders and stairs. Company visits took place at the company or at worksites on dates and times agreed on with the contact person of each company during a three-month period. Each visit consisted of a one- to two-hour interactive consultancy meeting with the contact person and workers of the construction company. Information was exchanged concerning selection, implementation and monitoring of safety measures with regard to rolling scaffolds, ladders and stairs. The safety consultant wrote a short report of the findings and the advised safety measures. In total, six safety consultants experienced in equipment for working from heights were involved in the face-to-face strategy.

#### Direct mail guidance strategy

The direct mail guidance strategy consisted of sending direct mail to the construction companies informing workers about the prevention of falling hazards with rolling scaffolds, ladders and stairs. The information consisted of a poster with URLs for the Internet approach (www.schilderenophoogte.nl) to four types of information and instruction materials: brochures and poster, checklists (instructions for safe installation and use of equipment), video, and a toolbox to inform and instruct workers during toolbox meetings.

### Measurements

#### Primary outcome measure

Safety violations were the primary outcome measure and were defined as the number and type of safety violations during the installation and use of equipment for working from heights. The labor inspectorate checked safety violations concerning equipment of rolling scaffolds, ladders and stairs on worksites of painters during a three-week period. The safety violations consist of 0 to 30 safety hazards [14].

#### Process measures

The following indicators for the implementation of the program were evaluated [16, 17]: reach, dose delivered, dose received. Satisfaction with the intervention (score 0–10), increase in perceived knowledge on safety measures (score 0–10) and perceived effectiveness on safety behavior (score 0–10) were used as indicators for acceptability concerning the interventions [18]. The process outcomes were measured during and after the intervention period by means of logbooks and questionnaires sent to the companies and their workers.

Reach was defined as the attendance rate of the construction companies at the intervention. Attendance rate was defined as the number of construction companies participating in this study relative to the number of eligible construction companies invited through the recruitment strategies. The attendance was assessed by means of a logbook during the recruitment of the construction companies.

Dose delivered referred to the proportion of the intended intervention that was actually delivered to the participating contact persons of the construction companies. For the face-to-face guidance strategy, the number of company visits and company reports including advice delivered by the safety consultants to the contact person of the construction companies was assessed by means of a logbook filled in by the safety consultants. For the direct mailing strategy, dose delivered was assessed by means of the number of direct postal mails and emails sent to the contact person of the construction companies. The dose delivered was rated as sufficient when more than 90 % of the companies received the interventions.

Dose received referred to the proportion of activities in the intervention that were actually performed by the employer and workers of the construction companies. For both intervention strategies, dose received was assessed by questions to the employer and workers after completion of the intervention period.

Satisfaction was measured by asking the employers and workers how they rated their satisfaction with the intervention as a whole and its individual components, on a scale from 0 (not satisfied) to 10 (very satisfied). Increased knowledge about working safely with rolling scaffolds, ladders and stairs was measured by asking the employers and workers to what extent their knowledge increased from (0–10; 0 = not more knowledge, 10 = much more knowledge). The rates were defined as not more knowledge (0), little (≥0 and <6), moderate (≥6 and <7.5) or much more knowledge (≥7.5) [14]. Effect on working safely with rolling scaffolds, ladders and stairs was measured by asking the employers and workers to what extent they rated the perceived effect on safety behavior (0–10, 0 = no effect, 10 = large effect). The rates were defined as poor (<6), moderate (≥6 and <7.5) or good (≥7.5).

The costs of the face-to-face strategy were calculated by multiplying the number of company visits by the fixed cost per visit, plus travel costs, coordination costs and the cost of information materials. The cost of the direct mailing strategy was calculated by totaling the coordination costs, the costs of developing information materials and the website, and the costs of direct mailing.

### Statistical analyses

At least 64 construction locations involved in painting and maintenance per group had to be captured to detect a reduction in safety violations of 15–25 % with an alpha of 0.05 (two-tailed) and a power of (1-beta) = 0.80. Differences in the mean number of safety violations and proportion of penalties were tested using Kruskal-Wallis one-way analysis of variance and Chi-Square tests respectively. The three process measurements were post-tested and differences between the interventions were examined using non-parametric tests. Statistical significance was defined as p < 0.05 for all outcome measures. The costs calculation for each intervention strategy was analyzed descriptively. The statistical analysis occurred after the pre-defined ending of the trial and after ascertainment of the required number of inspected companies (June 2014).

## Results

**Fig. 1:**
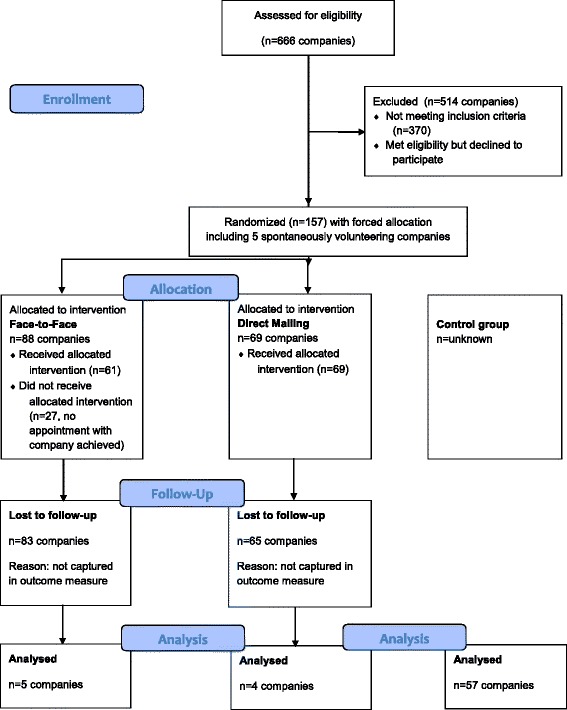
Overview of study design, number of participating and inspected companies

In total, 157 companies were included for participation to the face-to-face intervention or direct mail intervention. Figure 1 shows a flow diagram for the enrollment, allocation, follow-up and analysis of the construction companies for both intervention groups and control group.

In both intervention groups, 37 employers of companies responded to the questionnaire for the process measures dose received and perceived effectiveness (response rates face-to-face and direct mail, respectively 27 % and 19 %). Sixty-nine workers from 24 companies returned questionnaires: 45 workers from 15 companies in the face-to-face group and 24 workers from nine companies in the direct mail group. All process measures are presented below and summarized in Table 1.

**Table 1:** Overview of process evaluation measures on company level (reach and dose delivered), individual level among employers and workers (dose received, satisfaction, knowledge and safe work) and intervention level (costs)

		Face-to-face	Direct mail
Reach	Participating companies (%)	83/202 (41 %)	69/94 (73 %)
	Spontaneously volunteering companies	5	-
Dose delivered	Intervention activities delivered	61/88 (69 %)	69/69 (100 %)
Dose received	Employers shared information with workers	91 % (n = 21)	78 % (n = 9)
	Employers took action	88 % (n = 24)	62 % (n = 13)
	Workers received information	75 % (n = 44)	88 % (n = 24)
	Workers took action	62 % (n = 45)	58 % (n = 24)
Satisfaction (0–10)	Employers (mean, sd)	7.9 (1.27) (n = 22)	7.4 (0.88) (n = 9)
	Workers (mean, sd)	7.7 (1.07) (n = 34)	7.5 (0.98) (n = 21)
Increase in knowledge (0–10)	Employers (mean, sd)	5.7 (2.95) (n = 22)	6.6 (2.56) (n = 9)
	Workers (mean, sd)	7.5 (1.12) (n = 33)	6.8 (2.23) (n = 21)
Increase in safe work (0–10)	Employers (mean, sd)	6.8 (2.80) (n = 21)	6.8 (0.97) (n = 9)
	Workers (mean, sd)	7.5 (1.40) (n = 33)	6.9 (1.84) (n = 21)
Costs		€ 32,026	€ 25,000

### Reach

In total, 2188 painting companies were contacted by a call center requesting participation in one of the two interventions. With 666 companies, a structured interview was possible to determine the eligibility of the companies for this study, i.e. working in one of the 18 predetermined cities in the Netherlands during May 2014. With 1522 companies, no structured interview was possible due to the absence of a valid telephone number (729), not answering the telephone, or refusing to participate in the interview (793). Of those with completed interviews, 202 companies expected to work during the measurement period in the nine cities assigned to the face-to-face intervention; 94 companies expected to work during the measurement period in the nine cities assigned to the direct mail intervention.

In total, 41 % (83/202) of the eligible companies for the face-to-face intervention and 73 % (69/94) of the eligible companies for direct mail participated. Spontaneously, five companies were included in the group of personal advice after a request placed in a newsletter of the branch’s organizations (see Table 1). Reported reasons for not participating were: already sufficiently informed, safety sufficiently settled, no external advice, own professional workers, no time, no necessity.

### Interventions delivered and received

In the face-to-face group, 69 % (61/88) of the companies were visited by a safety consultant while 57 % (81/142) of the intended number of visits and 60 % (53/88) of the company reports were achieved. In the direct mail group, 100 % (69/69) received documentation (postal and email) while 52 % (36/69) visited the internet site and 13 % (9/69) did the safety test. In three companies, workers were allowed to be emailed directly (n = 10).

The majority of the employers reported that they provided feedback to their workers about safe working and had undertaken safety actions; in the face-to-face group, this proportion was higher than in the direct mailing group (91 % and 88 % vs. 78 % and 62 %). Additionally, the majority of the responding workers reported that they had been informed and had taken action.

### Satisfaction, perceived effectiveness and costs

Employers and workers were satisfied with the delivered intervention activities (means ranging from 7.4 to 7.9). Perceived increase in knowledge and safe work was rated between 5.7 to 7.5. No statistical differences between the interventions were found.

Costs for intervention activities for face-to-face intervention were €32,026 (safety consultants: 63 %; documentation materials 4 %; coordination: 33 %) and for direct mail intervention €25,000 (documentation materials and mailing: 49 %; website: 32 %; coordination: 20 %).

### Safety violations

Ultimately, nine intervention worksites of nine companies and 60 control worksites of 57 companies were captured in the 288 inspected worksites of painting companies. No statistical differences in mean number of safety violations and penalties were found between the intervention and control groups (see Table 2). The mean number of recorded violations and proportion of penalties varied between 1.8–2.4, respectively 72–100 %.

**Table 2:** Mean number of safety violations and proportion of penalties in intervention groups and control group

		Face-to-face	Direct mail	Control	*P*-value
Number inspected companies (worksites) by labor inspectorate		5 (5)	4 (4)	57 (60)^a^	
Penalty for companies		4 (80 %)	4 (100 %)	43 (72 %)	0.435
of which	…urging	-	1 (25 %)	4 (9 %)	
	…warning	4 (100 %)	2 (50 %)	24 (56 %)	
	…paying	-	1 (25 %)	7 (16 %)	
	…shutting down	2 (50 %)	1 (25 %)	17 (40 %)	
Mean number (SD) violations		2.4 (2.70)	1.8 (1.71)	1.9 (2.05)	0.847
Range number violations		0–7	0–4	0–8	

^a^In control group at three companies two independent worksites

## Discussion

The present randomized controlled intervention study evaluated two frequently advocated strategies to reduce safety violations in the construction industry and gain insight into their effect, implementation and acceptability. No conclusions about the effect of face-to-face and direct mail strategies on safety violations could be drawn due to the limited number of intervention companies captured in the primary outcome measurements. Perceived increase in knowledge and safe work did not differ between the intervention strategies. Financial costs for the face-to-face strategy were 28 % higher than the direct mail strategy.

### Methodological considerations

A strength of this national evaluation study was the double blinding in the assessment of the primary outcome measures. However, this advantage turned out to also be the major limitation of this study in terms of the low capture of intervention companies in the outcome measurements. Although the intended inspection sites by the labor inspectorate–based on power analyses and expected loss to follow-up and inspection of non-painting construction sites–were achieved (in total 288 worksites: 122 worksites in cities assigned to face-to-face intervention group, 106 worksites in cities assigned to direct mailing intervention group and 60 worksites assigned to cities in control group), only nine intervention companies were captured in the outcome measurements by the labor inspectorate. Due to ethical reasons, i.e. introducing unequal risk of incurring financial penalties when safety violations were established by the labor inspectorate, all construction sites in the 27 cities were able to be the subject of inspection and the researchers could not reveal the names of the 18 intervention cities nor the names of the participating painting companies. Intervention research in the construction industry remains a challenge because of the many workplace factors prevalent in the construction setting that may need to be accounted for in research design [19]. Among others, a jobsite moves from place to place, and most construction employees work on several jobsites each year, while placing bids for work often do not include specification requirements for health and safety equipment and procedures [19].

Furthermore, the program implementation in terms of dose delivered and dose received was insufficient. It was difficult to make appointments with the construction companies for worksite visits in the group of companies with the face-to-face strategy. Although in the companies with the email strategy dose delivered in terms of sending information was guaranteed, the uptake of information was low in terms of consulting the internet or testing the knowledge.

The practice base has been incorporated to the maximum in both interventions [14]. For both guidance strategies, the content and approach has been developed by the Dutch institute on safety and health in the construction industry. The safety consultants were experienced in safety in construction work and the information materials were adapted to the context of painters. In addition, construction companies were free to choose how they wanted to control the safety hazards when working with rolling scaffolds, ladders and stairs. Possibly, this practice base in combination with the recruitment strategy with structured telephone interviews [14] contributed to the high recruitment rates for the face-to-face interventions and the direct mail strategy with much higher rates than reported in other intervention studies in the construction industry [20, 21], and especially for small construction companies in voluntary safety programs or safety research [22]. Possibly due to the fact that there was no need to make further appointments in the direct mail strategy compared with the face-to-face intervention, the direct mail intervention achieved the highest recruitment rate of over 70 %.

A strength in this design was the process measurements that gave insight into the acceptance and perceived increase in knowledge and safe work among employers and workers. However, due to *a priori* agreed restrictions in approaching the companies for research goals, process measures could not be evaluated in more detail, e.g. performed safety measures and involved number of workers. Many reviews on intervention studies stipulate the importance of process evaluations alongside the effect evaluations [23].

### Practice implications

In both interventions, three important implementation measures were assessed: knowledge in safe working (awareness); satisfaction and perceived safety (attitude); and safety violations (behavior). Both among employers and workers, increase in knowledge was rated as moderate for the direct mail strategy; for the face-to-face strategy, increase in knowledge was rated as low among employers and high among workers. This lower rate among employers for the face-to-face strategy could be due to higher expectations regarding personal knowledge transfer. Since attitude in terms of perceived increase in safe work and satisfaction were moderate to high in both strategies among employers and workers, both strategies are acceptable for stimulation of safety behavior.

Possibly, additional strategies for the stimulation of personal feedback could be built in for internal organized feedback in both the face-to-face strategy and direct mail approach. An example could be the coaching of construction site foremen to include safety in their daily verbal exchanges with workers that showed a positive and lasting effect on the level of safety [24].

The effect on actual safety behavior in terms of safety violations did not differ between intervention and control groups, but–as stated in the methodological considerations–this result is biased due to the low number of intervention companies in the outcome measurements. However, the mean number of violations and penalties did not differ between the nine captured intervention companies and the other 279 inspected companies. Consequently, there are no indications of any effect of the interventions on safety violations. The high prevalence of penalties at inspected construction sites underlines the need to counteract unsafe working with rolling scaffolds, ladders and stairs.

Finally, the costs for the face-to-face strategy are 28 % higher compared with the direct mail strategy. Taking into account the difference in actual delivered implementation, this difference increases from €250 to €525 per company.

### Lessons learned

From a methodological point of view, two lessons can be learned, namely the efficient and successful procedure of recruitment of companies and the lack of data triangulation of the primary outcome measure. Firstly, many eligible companies were identified through a call center, and an adequate number of companies were willing to participate. In many studies, especially in the construction industry, the recruitment phase takes a long time. Secondly, allowing researchers extra effort in visiting the intervention companies to assess the primary outcome measure–besides the measurements by the labor inspectorate–would have resulted in a sufficient number of analyzed companies, thereby making it possible to answer the first research question. Unfortunately, a limited number of intervention companies were actually working in the assigned cities during the follow-up measurement period specified by the labor inspectorate, probably due to the short and alternating duration of many construction projects. Therefore, choosing inspector assessment as outcome measure alone resulted in too few assessments of intervention companies. This might be overcome by another study design, e.g. more qualitative and explorative designs alongside a thorough process evaluation. For example, in-depth interviews with employers and workers during the intervention could provide insight into barriers and facilitators for increasing the intervention uptake. Also, more participatory approaches with active involvement of researchers could increase insight into the intervention process.

From a practical point of view, one main lesson has been learned, namely the low intensity of the delivered and received interventions due to variable work settings at construction sites. Since behavioral change requires continuous attention and feedback to tackle barriers at organizational and worksite level, e.g. appointments with suppliers of rolling scaffolds or using instructions when setting up and using climbing materials.

## Conclusions

No conclusions about the effect of face-to-face and direct mail strategies on safety violations could be drawn due to the limited number of intervention companies captured in the primary outcome measurements. The costs for a face-to-face strategy are higher compared with a direct mail strategy. No difference in awareness and attitude for safe working was found among employers and workers between both strategies.
